# Research Progress on Mechanisms of Milk-Derived Functional Components in Regulating Bone Health and the Gut–Bone Axis

**DOI:** 10.3390/nu18182964

**Published:** 2026-09-10

**Authors:** Henigul Osman, Habiram Hamit, Yating Wu, Shiqi Zhang, Xianlan Ma, Hongyan Zhang, He Chen, Yankun Zhao

**Affiliations:** 1 Institute of Quality Standards & Testing Technology for Agricultural Products, Xinjiang Academy of Agricultural Sciences, Urumqi 830091, China; 13009693320@163.com (H.O.); 15199796181@163.com (H.H.); wuyating0223@163.com.com (Y.W.); zhangshiqi1993@xaas.ac.cn (S.Z.); 320242735@stu.xjau.edu.cn (X.M.); zhanghongyan1978@xaas.ac.cn (H.Z.); chenhe_1971@163.com (H.C.); 2 Key Laboratory of Functional Nutrition and Health of Characteristic Agricultural Products in Desert Oasis Ecological Region (Co-Construction by Ministry and Province), Ministry of Agriculture and Rural Affairs, Urumqi 830091, China

**Keywords:** milk-derived active ingredients, osteoporosis, bone metabolism, “gut–bone” axis, functional food

## Abstract

Osteoporosis affects over 200 million people worldwide, with postmenopausal women at the highest risk. The treatment benefits of currently available drugs are constrained by safety concerns, and they lack bidirectional regulation of bone metabolism, driving growing interest in safe dietary interventions. Milk is rich in functional components, including minerals (calcium, phosphorus), casein phosphopeptide (CPP), lactoferrin (LF), osteopontin (OPN), and milk fat globule membrane (MFGM), that promote mineral absorption, regulate bone cell activity, and support intestinal health. This review examines their dual mechanisms in bone health regulation. Directly, LF activates Wnt/MAPK pathways to enhance osteogenesis and inhibit osteoclast activity, OPN modulates mineralization, and CPP synergizes with calcium and phosphorus to support bone formation. Indirectly, these components modulate gut microbiota via the gut–bone axis, raising short-chain fatty acids, lowering intestinal pH to boost calcium absorption, repairing the intestinal barrier, and reducing systemic inflammation. Remaining challenges lie in extraction, purification, and product development. Looking forward, the field must move from cell and animal-level evidence to human clinical validation and establish robust steady-state delivery of these bioactives. Successful translation along these lines is essential to turn milk-derived components into effective, evidence-based functional foods for bone health.

## 1. Introduction

Bone serves as the fundamental structural framework that maintains the integrity of the organism and supports motor function, and its metabolic homeostasis depends on a delicate and tightly regulated balance between bone formation mediated by osteoblasts and bone resorption mediated by osteoclasts [1]. With advancing age, the body commonly undergoes physiological changes characterized by progressive bone loss, deterioration of bone microarchitecture, and inadequate intake of nutrients essential for bone metabolism, such as calcium and vitamin D. Moreover, long-term adverse lifestyle factors, including smoking and excessive alcohol consumption, can further accelerate these processes and exacerbate bone loss [2,3]. Against the backdrop of a steadily aging global population, the prevalence of metabolic bone disorders such as osteoporosis has been rising year by year, with correspondingly increasing fracture burden and healthcare-related economic costs.

Osteoporosis is a bone metabolic disease characterized by decreased bone mass, degraded bone microstructure, and decreased bone mineral density. Its occurrence is often closely related to factors such as decreased estrogen levels and calcium metabolism disorders [4]. According to the International Osteoporosis Foundation, about 200 million people around the world are affected by osteoporosis. About one-third of women and one-fifth of men over the age of 50 have at least one fracture in their lifetime. It is reported that the prevalence of osteoporosis in people over 50 years old in China is 19.2%, of which women (32.1%) are significantly higher than men (6.0%), and the prevalence among women is about 5 times that of men. The prevalence rate in people over 65 years old increased to 32.0%, and that of women was as high as 51.6% [5]. Further studies have shown that the absolute burden of osteoporosis in menopausal women continues to increase, with the highest risk in low-and middle-income countries and women over 85 years of age [6]. According to regional statistical data, in Canada, Europe, and the United States alone, the direct medical costs of osteoporotic fractures amount to 500–650 billion USD annually. When indirect economic costs such as disability and lost productivity are further taken into account, the socioeconomic burden imposed by this disease increases substantially, and its impact should not be underestimated [7].

At present, there are many drugs, such as alendronate sodium, denosumab, salmon calcitonin and raloxifene, used in the clinical treatment of metabolic bone diseases such as osteoporosis. These drugs have the characteristics of high cost-effectiveness and medicinal value, convenient use, effective improvement of bone mineral density, and reduction of fracture risk. However, although these drugs are widely used in clinical practice to prevent and treat osteoporosis, long-term use is often accompanied by adverse reactions such as jaw necrosis and rebound bone loss. Toriumi et al. [8] pointed out in their study that bisphosphonates, denosumab, and over a dozen other drugs are significantly associated with osteonecrosis of the jaw, and the underlying predisposing factor lies in the effect of these medications on osteoclast activity. Tsourdi et al. [9] showed that rapid bone loss and multiple vertebral fractures occurred in most patients after the withdrawal of denosumab. More importantly, the above drugs are mostly targeted at a single regulation of bone metabolism, making it difficult to effectively maintain the dynamic balance between osteogenesis and osteoclasts. Therefore, the development of natural and safe milk-derived functional components to regulate bone metabolism balance has become a research hotspot in this field.

Milk and dairy products are natural foods with comprehensive nutrition. In addition to basic nutrients such as proteins and minerals, they also contain a variety of functional components with special biological activities [10]. Taking calcium as an example, animal milk is rich in calcium and has a high absorption rate, whereas the presence of antinutritional factors such as oxalic acid and phytic acid in plant-based milk interferes with calcium absorption, resulting in a lower absorption rate [11]. Therefore, this paper elucidates the whole chain mechanism of milk-derived functional components from “intestinal absorption” to “bone tissue action” and discusses the differential advantages of different active components in order to provide a theoretical reference for promoting the development and application of milk-derived functional components in the field of bone health.

## 2. Methods

This narrative review identified the relevant literature through a comprehensive search of the Web of Science, PubMed, and CNKI databases, covering publications from the past decade. The search terms included “milk-derived components”, “casein phosphopeptide”, “lactoferrin”, “osteopontin”, “bone health”, “osteoporosis”, “gut-bone axis”, and “short-chain fatty acids (SCFAs)”. As a narrative review, the literature was collected with an emphasis on expert-based synthesis, aiming to provide an integrative overview of this field. Included studies were original research articles or reviews that addressed the role of milk-derived bioactive components in the regulation of bone metabolism. Reference lists of the included articles were manually screened to identify additional relevant studies, with a particular focus on publications between 2017 and July 2026. After stepwise screening, the final set of eligible articles was selected for full review; among these, 83 were published within the last five years, and 80 within the last three years.

The final included literature was categorized and analyzed according to two conceptual frameworks: (1) the direct pathway, involving bone formation and resorption mediated by osteoblasts and osteoclasts; and (2) the indirect pathway, involving modulation through the gut–bone axis and microbial metabolites such as short-chain fatty acids.

## 3. The Key Functional Components Regulating Bone Health in Milk Sources and Their Mechanism of Action

**Figure 1:**
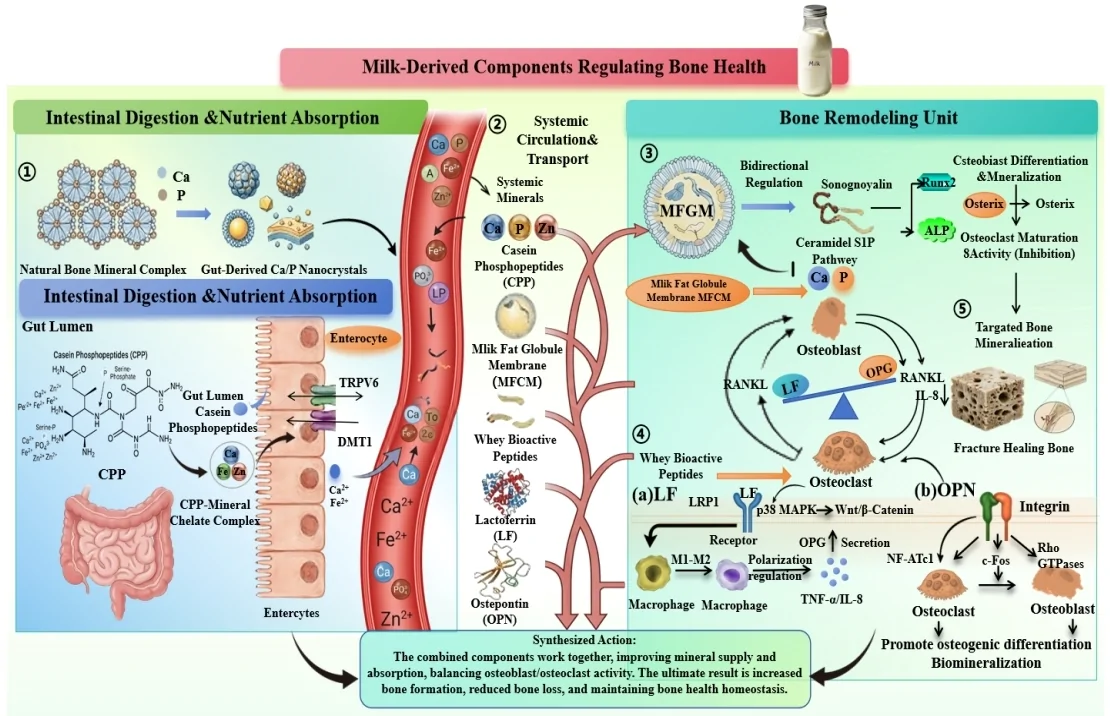
Mechanism of milk-derived functional components in regulating bone health.

The currently identified milk-derived bioactive components, such as natural calcium–mineral complexes, casein phosphopeptides (CPP), milk fat globule membrane (MFGM), and lactoferrin (LF), as well as osteopontin (OPN), have been shown to have a positive effect on bone health. However, previous studies have been limited to treating them as simple nutritional supplements or anti-inflammatory factors, lacking a systematic interpretation of their molecular regulatory network and the synergistic interactions among these bioactive components. In view of this, this section deeply analyzes the dual regulation mechanism of the above components, spanning intestinal absorption and the bone remodeling microenvironment, based on Figure 1. On the one hand, it elucidates the metabolic pathway for efficient mineral absorption mediated by CPP. CPP forms chelate complexes with calcium-zinc minerals in the intestinal lumen and facilitates mineral uptake through intestinal-specific transporters (TRPV6, DMT1), providing adequate mineral substrates for bone biomineralization. On the other hand, it focuses on revealing how these components coordinate with one another after entering systemic circulation. MFGM exerts sphingomyelin-ceramide/S1P-dependent regulation on bone cells; MFGM and LF synergistically inhibit the RANKL/NFATc1 osteoclast signaling axis and activate the Wnt/β-catenin osteogenic pathway. Meanwhile, LF cooperates with OPN to fine-tune the RANKL-OPG balance axis, and OPN further amplifies osteogenic differentiation and biomineralization via integrin-Rho GTPases signaling. Such upstream-downstream cooperation, where CPP supplies bioavailable minerals and MFGM/LF/OPN execute local bone signal modulation, achieves precise regulation of bone homeostasis. This change in perspective indicates that our understanding of the function of milk-source components is moving from the traditional “nutritional supplement” to deeper “molecular signal intervention”.

### 3.1. Milk-Derived Mineral Elements: Natural Bone Mineral Complex

Milk-derived minerals are naturally occurring inorganic nutrients in milk, which are rich in potassium (K), sodium (Na), calcium (Ca), phosphorus (P), iron (Fe), copper (Cu), zinc (Zn), selenium (Se), iodine (I), manganese (Mn) and other elements [12]. Among them, Ca and P are the most abundant, and most of these minerals bind to casein micelles to form amorphous nano-calcium phosphate colloidal complexes (also known as calcium phosphate nanoclusters or micelle calcium phosphate, MCP) [13,14]. Huppertz et al. [13] showed that the dairy product matrix encapsulates MCP through casein micelles and slowly releases it, and does not contain anti-nutritional factors (phytic acid, oxalate, etc.) that hinder calcium absorption, thereby significantly improving the bioavailability of calcium. He et al. [15] found that milk calcium or calcium carbonate can regulate the composition of intestinal microorganisms and their digestive and metabolic activities, significantly increasing bone mineral density (BMD) and femoral mechanical strength, and further confirming the correlation of the “gut–bone” axis.

In bone metabolism, extracellular calcium ions and inorganic phosphates are key factors that determine bone mineralization [16]. The bone matrix is mainly composed of type I collagen, non-collagen protein and calcium phosphate mineral components, and its synthesis rate and mineralization degree directly determine bone mass and bone strength [17]. Ensuring adequate calcium intake in early childhood can effectively promote bone mineralization and healthy development and help achieve ideal bone mineral density and bone strength [18]. Farag et al. [19] showed that children under one year of age require a daily supplemental intake of approximately 200–260 mg of calcium, 100–275 mg of phosphorus, and vitamin D (400 IU/day); failure to meet these nutritional requirements may increase the risk of bone metabolic disorders due to inadequate mineral supply. The aforementioned mineral elements synergistically improve the bone nutritional structure from multiple dimensions, such as bone synthesis, bone microstructure maintenance and bone protection. Therefore, milk-derived minerals are a kind of natural bone nutrient source with high safety and high bioavailability.

### 3.2. Casein Phosphopeptide (CPP): Mineral Element Chelation, Absorption Promotion and Synergistic Osteogenesis

Casein (CN) is the most abundant phosphorylated protein in milk, accounting for about 80% of total milk protein [20]. Casein phosphopeptide (CPP) is a kind of phosphorylated active peptide produced by the enzymatic hydrolysis of casein [21]. Its core active structure consists of three phosphorylated serines and two glutamic acids (-SerP-SerP-SerP-Glu-Glu-) [22]. This structure endows CPP with the ability to form soluble chelates with divalent cations (such as Ca^2+^, Fe^2+^, Zn^2+^, etc.) [23]. Studies have shown that CPP can efficiently bind to calcium ions under intestinal acidic and alkaline conditions, and significantly improve the bioavailability of calcium [24]. Its mechanism is mainly reflected in two aspects. On the one hand, Tenenbaum et al. [25], through in vitro simulated gastrointestinal digestion experiments, confirmed that CPP after digestion can up-regulate the gene expression level of the TRPV6 calcium channel protein, thereby promoting transepithelial calcium transport. On the other hand, Sanjulián et al. [26] pointed out that CPP can bind to calcium ions in the low pH environment of the gastrointestinal tract, effectively preventing the formation of calcium phosphate precipitation, thereby improving the bioavailability of calcium and phosphorus.

In terms of bone metabolism regulation, CPP can improve the solubility and absorption efficiency of calcium in the intestine by efficiently chelating mineral elements such as calcium and phosphorus. At the same time, it cooperates with these mineral elements to activate osteoblasts, reduce bone resorption, and increase bone density, thereby positively regulating bone metabolism. Clinical studies have shown that CPP formula milk powder can effectively promote calcium absorption and have a positive effect on bone mineral density index and bone health during the growth period [17]. Reddi et al. [27] found that casein-derived peptides significantly increased bone density by reducing the expression levels of Receptor Activator of Nuclear Factor-κB Ligand (RANKL), Interleukin-6 (IL-6) and Tumor Necrosis Factor-α (TNF-α), and had a positive effect on bone. In addition, Lee et al. [28], through in vitro experiments, confirmed that MDP-DOPA scaffolds prepared from bovine casein can induce anti-inflammatory M2 macrophage polarization, and when combined with CPP, they can significantly promote the osteogenic differentiation of bone marrow mesenchymal stem cells (BMSCs). In the field of oral health, CPP also shows application value. Casein phosphopeptide–amorphous calcium phosphate (CPP-CAP) added to food (such as candy, gum, milk) has a dual role in anti-demineralization and promoting the remineralization of enamel [29]. Bianca et al. [30,31] found that the combination of CPP-CAP and fluoride can effectively reduce the degree of white spot lesions associated with dental caries. In addition, the combination of Er, Cr:YSGG laser and CPP-ACP can also significantly reduce white spot lesions, and the use of laser alone can also reduce enamel demineralization [32].

### 3.3. Milk Fat Globule Membrane (MFGM) and Its Active Lipids

Milk Fat Globule Membrane (MFGM) is a layer of membrane structure with special biological activity, naturally wrapped on the surface of mammalian milk fat globules [33], and its thickness is generally between 10 and 50 nm [34]. MFGM is mainly composed of active components such as phospholipids, sphingolipids, and glycoproteins [35]. Among them, sphingolipids are the most important component in MFGM and an important component of mammalian plasma membrane lipids [36].

It has been reported that sphingomyelin and its metabolites play an important role in the regulation of bone metabolism. Phosphatidylcholine (PC) and sphingomyelin (SM) play key regulatory roles in bone formation and mineralization [37]. Qi et al. [38] showed that sphingomyelin, sphingomyelin-1-phosphate (S1P) and other sphingolipid metabolites can regulate the differentiation and activity of osteoblasts and osteoclasts in a two-way manner, thus maintaining the dynamic balance of bone more precisely. On the contrary, the disorder of sphingolipid metabolites may lead to bone metabolic diseases and affect the healthy development of bone. Grewe et al. [39] showed that S1P, as a coupling factor between osteoclasts and osteoblasts, can effectively promote the proliferation and differentiation of osteoblasts, but an imbalance in S1P levels may increase osteoporosis-related fracture risk. Armstrong et al. [40] found that the intake of whey protein phospholipid concentrate (WWPC) significantly increased the calcium absorption rate of mice by regulating the intestinal flora (*Bifidobacterium*), thus having a positive effect on bone health. Ma et al. [41] found that long-chain polyunsaturated fatty acids (such as docosahexaenoic acid, DHA) reduced the expression of RANKL secreted by osteoblasts by inhibiting the phosphorylation of ERK1/2 and JNK, thereby inhibiting the formation of osteoclasts. In summary, MFGM and its active lipids can regulate the metabolic activity of bone and have a positive effect on bone health.

### 3.4. Whey Active Peptide

Whey protein peptide is a kind of small-molecule active peptide produced by the enzymatic hydrolysis or fermentation of whey protein, which shows many biological activities such as antihypertensive (ACE inhibitory peptide), antioxidant, antibacterial and anti-tumor [42]. In the regulation of bone metabolism, whey protein peptides synergistically stimulate osteoblast proliferation and inhibit osteoclast activity through multiple mechanisms, thereby reducing bone loss and promoting bone formation. Nielsen et al. [43] showed that YVEEL and YLLF peptides derived from bovine *β-lactoglobulin* have significant bone-protective activity by reducing the levels of inflammatory cytokines and increasing bone formation markers.

Lactoferrin-derived peptides also show potential for bone protection. Studies have shown that lactoferrin-derived peptides can regulate the activation of the STAT3 signaling pathway stimulated by interleukin-11 to protect chondrocytes. At the same time, they can also activate the p38/MAPK, PI3K/AKT and IGF-R1 signaling pathways to achieve anti-inflammatory effects, osteogenic differentiation and mineralization, inhibition of osteoclast formation and activity, etc. These peptides play a significant role in the treatment of orthopedic diseases and bone tissue repair [44]. In addition, in a high-fat diet-induced obese mouse model, whey protein hydrolysate effectively improved bone loss and bone microstructure degradation by activating the GSK-3β/Nrf2 signaling pathway and up-regulating the expression levels of Runx2, Nrf2 and HO-1 proteins [45].

### 3.5. Lactoferrin (LF) and Osteopontin (OPN): Bone Immune Regulation and Signaling Pathway of Lactoglycoprotein

Lactoferrin (LF) is an iron-binding glycoprotein that belongs to the transferrin family. It is mainly present in the external secretions of mammals and has the highest content in colostrum [46]. From the perspective of structure, LF has a typical spherical conformation, and its polypeptide chain is coiled and folded to form two symmetrical leaf-like domains. Each domain can reversibly bind and transport a portion of iron ion (Fe^3+^) [47], exhibiting a complete molecular structure and good iron ion chelating ability [48]. In physiological processes, LF is a multifunctional regulator that maintains intestinal health. Its main functions include promoting intestinal development, regulating iron absorption, participating in immune regulation, and maintaining bacterial balance [49].

In the regulation of bone metabolism, LF has a wide range of physiological activities on bone cells. Studies have found that both human lactoferrin (hLF) and bovine lactoferrin (bLF), when treated with different methods, can promote the differentiation of MC3T3-E1 cells and enhance their mineralization ability [50]. Nagashima et al. [51] compared the effects of recombinant hLF and bLF on osteogenic differentiation using MC3T3-E1 cells as a model. The results showed that the two had similar effects; they not only significantly promoted osteoblast differentiation, but also significantly up-regulated the expression levels of alkaline phosphatase (ALP) and osteopontin (OPN). Sun et al. [52] found that LF enhanced the ALP activity of MC3T3-E1 cells under mechanical strain and increased the expression levels of p-Runx2 and p-ERK1/2 in MC3T3-E1 cells, which effectively promoted the proliferation, differentiation and mineralization of osteoblasts.

At the signaling pathway level, LF can regulate the physiological activity of bone cells through specific receptor pathways and activate Wnt, MAPK, PI3K/Akt and RANK/RANKL/OPG signaling pathways, thereby promoting osteoblast proliferation and differentiation, accelerating bone collagen synthesis, and promoting bone matrix formation; at the same time, it reduces the rate of bone resorption and bone loss, and achieves a two-way balance of bone metabolism. In the osteogenic regulatory network, the Wnt/β-catenin pathway is at the core. It can cooperate with bone morphogenetic protein (BMP) to enhance osteogenic differentiation, and also with transforming growth factor-β (TGF-β), as well as Hippo and Notch signaling pathways to regulate the differentiation direction and osteogenic activity of bone marrow mesenchymal stem cells [53]. Fan et al. [54] have shown that a microgravity environment can inhibit the Wnt/β-catenin pathway by down-regulating FAK phosphorylation levels, thus inhibiting bone formation. In addition, Khotib et al. [55] found that the regulation of ERK, p38, Wnt and BMP2 pathways can affect osteoblast differentiation under the intervention of hydroxyapatite materials. The above results together indicate that Wnt/β-catenin and its related pathways are key nodes in the regulation of bone metabolism and provide a theoretical basis for the osteogenic mechanism of LF.

In addition, LF plays an important regulatory role in the bone immune microenvironment. Macrophages are the core innate immune cells in the this microenvironment, and their M1 (pro-inflammatory)/M2 (anti-inflammatory) polarization balance directly determines the level of tissue repair [56]. LF can induce the polarization of macrophages from pro-inflammatory M1 to anti-inflammatory M2, thereby improving the bone immune microenvironment [57]. In pathological conditions such as osteoporosis or bone defects, the enrichment of pro-inflammatory factors such as Interleukin-12 (IL-12), Interferon-γ (IFN-γ), Lipopolysaccharide (LPS) continues to induce M1 polarization, resulting in enhanced osteoclast activity, accelerated degradation of the bone matrix, and inhibition of BMSCs osteogenic differentiation, which hinders bone repair [58,59,60]. Zhou et al. [61] found that LPS could inhibit the differentiation of osteoblasts in a dose-dependent manner, up-regulating the expression of OPG and down-regulating the expression of RANKL in osteoblasts through TLR4, which indirectly reduced osteoclast formation. LF effectively reverses this imbalance by down-regulating the expression of the pro-inflammatory factor IL-6 and stimulating the release of anti-inflammatory factors Interleukin-4 (IL-4) and Interleukin-10 (IL-10), thereby reducing the damage of inflammation to bone tissue and creating a microenvironment conducive to bone repair [57].

However, the reported effects of LF are not entirely consistent across studies. Although multiple lines of evidence indicate that LF is a potent osteoblast mitogen that inhibits osteoclast development, it reportedly fails to alter the resorptive function of mature osteoclasts, implying distinct mechanisms governing osteoclastogenesis versus mature osteoclast activity. Notably, such discrepancies are amplified by methodological heterogeneity, particularly the iron-saturation state of LF, ranging from apo- (iron-free, <5%) to holo-instances (iron-saturated, >85%)—which profoundly affects its activity [57,62]. The apo-, mono- and holo-forms exhibit distinct functional profiles [63,64], and this variability rather than LF per se may underlie conflicting observations [65], given that iron itself modulates osteoclast differentiation and bone resorption. Nevertheless, apo-LF has been shown to confer antibacterial activity (reduced upon iron saturation), while its immunomodulatory actions, such as inhibiting granulocyte-macrophage colony-stimulating activity and neutrophil migration, are likewise iron-saturation-dependent [66,67].

Osteopontin (OPN), also known as milk bridge protein, is a secreted phosphorylated glycoprotein naturally present in the bone matrix, immune cells and various tissues [68]. OPN from different sources, including human milk, bovine milk and goat milk, has a high degree of similarity in amino acid sequence and biological function. OPN has a variety of biological functions. In addition to participating in the regulation of inflammatory response, it also has the function of biomineralization [69]. It has been reported that OPN is a high content of non-collagen protein in milk, which can affect enamel remineralization and reduce the risk of dental caries [70]. In addition, Zhang et al. [71] found that compared with the use of LF and OPN alone, the combination of LF and OPN can activate the Notch signaling pathway by up-regulating the expression level of Brg1, which is more effective in promoting the maturation of the intestinal barrier and improving its structural integrity in neonatal mice. Studies have also confirmed that the complex formed by LF and OPN (iron-free LF and calcium-saturated OPN, namely apo-LF and holo-OPN) can significantly enhance the proliferation activity of intestinal epithelial cells by regulating the PI3K/Akt signaling pathway, thereby further improving intestinal function [72].

In terms of bone health, the functions of OPN should be viewed from two distinct perspectives: as a structural bone matrix protein and as a signaling molecule. As a structural component, OPN is a major noncollagenous bone matrix protein that participates in matrix organization and calcium deposition, thereby regulating bone mineralization and maintaining the toughness and stability of bone structure. As a signaling molecule, OPN mediates the adhesion, proliferation and differentiation of osteoblasts and osteoclasts through its RGD cell-attachment sequence, which binds αvβ3 integrin, and through its calcium-binding and heparin-binding domains, which interact with CD44. Through these dual roles, OPN can accurately regulate the rate of bone mineralization, avoid over-mineralization or under-mineralization, and maintain bone structural integrity. Some studies have shown that OPN supplementation may have a positive impact on bone health by regulating bone metabolism markers (such as osteogenesis-related factors and osteoclast-related factors) [73,74]. In the process of mineralization, OPN participates in the deposition of calcium salts in vivo and shows significant osteogenic activity. In terms of mechanism, OPN can inhibit osteoclast activity and reduce bone resorption by down-regulating transforming growth factor-β (TGF-β) [75]. Other studies have shown that OPN itself does not directly inhibit bone resorption, but indirectly inhibits osteoclast activity by knocking out or down-regulating OPN to reduce the expression level of miR-34c [76]. In the intestine, OPN has excellent intestinal repair and immune regulation ability, which can repair the intestinal epithelial barrier and promote the proliferation of beneficial bacteria. Lang et al. [77] have shown that OPN plays a key role in maintaining intestinal barrier function, which can alleviate tissue damage caused by chronic inflammation by promoting epithelial cell survival and enhancing mucosal regeneration.

However, the literature on osteopontin is not entirely consistent, particularly regarding whether OPN genuinely supports resorptive activity. While most studies support a role for OPN in promoting osteoclast adhesion and migration—primarily via αvβ3 integrin and CD44 receptors—a central methodological issue is posttranslational modification (PTM). OPN is a multiply phosphorylated glycoprotein, and its extensive PTMs, including phosphorylation and glycosylation, markedly influence its structure and function. More broadly, PTMs such as phosphorylation are recognized as critical regulators of bone formation, bone resorption, and the osteoclast-mediated resorption machinery [78]. Critically, evidence indicates that OPN PTMs, possibly phosphorylation, are required for in vitro bone resorption but not for osteoclast adhesion [79]. In line with this, dephosphorylation of OPN (and bone sialoprotein) by osteoclast-derived tartrate-resistant acid phosphatase (TRACP) abolishes osteoclast adhesion to the substrate [80]. Consequently, conclusions regarding OPN’s effects on osteoclasts depend substantially on the isoform and modified state of the protein studied and on the specific functional readout selected (e.g., adhesion versus resorption-pit formation).

### 3.6. Modulatory Effects of Milk-Derived miRNAs on Bone Formation

In recent years, microRNAs (miRNAs) have garnered increasing attention as key factors in the nutritional regulation of bone formation. As a class of small non-coding RNAs, miRNAs mediate the degradation and translational repression of target mRNAs by binding to the seed sequences within their 3′-untranslated regions (3′-UTRs), thereby exerting bidirectional regulatory effects on bone formation-related signaling pathways, including bone morphogenetic protein (BMP), Wnt/β-catenin, Notch, and TNF-α signaling [81]. It has been reported that exercise and dietary nutrition are important modulators of bone metabolism, primarily through epigenetic regulation via alterations in miRNA/miR expression, thereby preventing or delaying bone metabolism-related diseases [82]. Proia et al. [83] found that running stimulates the expression of miR-21–5p, which relieves its inhibitory effects on PTEN and Smad7. Downregulation of PTEN activates the PI3K/AKT/mTOR pathway, promoting osteoblast proliferation and differentiation; meanwhile, downregulation of Smad7 relieves its antagonistic effect on the TGF-β/SMAD signaling pathway, consequently upregulating RUNX2 expression and accelerating osteogenic differentiation. miR-218–5p has been shown to promote mineralized nodule formation, increase alkaline phosphatase (ALP) activity, and elevate the levels of osteogenic markers such as ALP, RUNX2, and BMP-2.

More critically, bovine milk exosomes are enriched in multiple miRNAs, among which miR-148a-3p has been demonstrated to promote osteogenesis [84]. Previous studies have confirmed that dietary supplementation with whey protein concentrate rich in bovine milk exosomes can promote longitudinal growth and maintain trabecular bone microarchitecture in vivo, contributing to enhanced longitudinal bone growth in rats and supporting skeletal health during catch-up growth [85]. Moreover, milk-derived small extracellular vesicles may exert dual protective effects by promoting bone formation and alleviating inflammation within inflammatory microenvironments, such as chronic apical periodontitis, potentially through the regulation of miR-21 [86]. Collectively, dietary-derived miRNAs possess the potential to modulate bone formation. As a novel class of regulatory factors in this field, they may be harnessed through the synergistic effects of exercise and nutrition to modulate the miRNA network, offering a safe and effective strategy for promoting skeletal health and preventing metabolic bone diseases such as osteoporosis.

This section systematically reviews the mechanisms by which milk-derived bioactive components regulate bone metabolism through direct cellular signaling pathways. Milk-derived bioactive components regulate bone metabolism through both direct and indirect cellular signaling pathways. Directly, micellar calcium phosphate (MCP) provides a bioavailable, sustained-release basis for mineralization; casein phosphopeptides (CPPs) promote transepithelial calcium transport via TRPV6, activating osteoblasts while downregulating RANKL, IL-6 and TNF-α; whey- and lactoferrin-derived peptides drive osteogenic differentiation through p38/MAPK, PI3K/Akt and GSK-3β/Nrf2; and core glycoproteins lactoferrin (LF) and osteopontin (OPN) promote bone formation and inhibit resorption via Wnt/β-catenin, MAPK, PI3K/Akt and RANK/RANKL/OPG, while reshaping the bone immune microenvironment by inducing M1-to-M2 macrophage polarization. Indirectly, MFGM sphingolipids and metabolites such as S1P couple osteoblast–osteoclast crosstalk, while OPN modulates mineralization and bone homeostasis via TGF-β, miR-34c and intestinal barrier function. However, key questions remain unresolved: whether LF’s conflicting effects on osteoclasts reflect genuine context-dependence or preparation/dosage artifacts (given the apo- vs. holo- iron-saturation difference and iron’s own modulation of osteoclast differentiation); how OPN’s PTM state governs its resorptive versus adhesive function; and how LF and OPN jointly modulate osteoclast function, which remains underexplored. Addressing these questions will be essential for translating these findings into clinical benefit.

## 4. The “Gut–Bone” Axis: A Systematic Pathway for Milk-Derived Components to Regulate Bone Health

**Figure 2:**
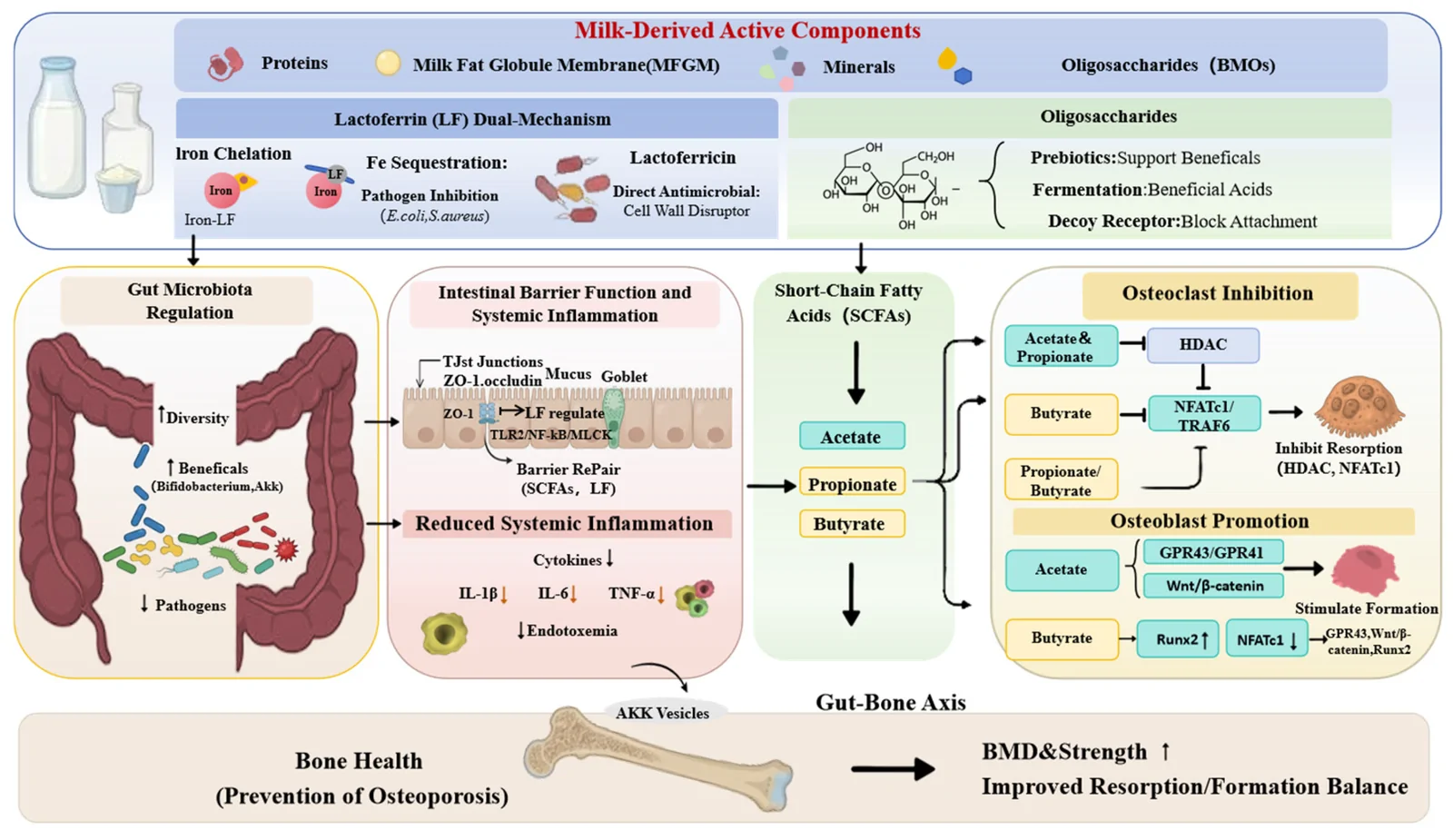
Molecular mechanisms by which milk-derived bioactive components regulate bone homeostasis through the gut microbiota-short-chain fatty acid signaling pathway.

As the cornerstone of mechanical support and the central hub of calcium–phosphorus metabolism, the skeleton plays a vital role in maintaining quality of life throughout the human lifespan. Recent studies have demonstrated that the protective effects of dairy products on bone health extend far beyond mere calcium supplementation, relying instead on the synergistic actions of milk-derived bioactive components (such as LF, OPN, CGMP, and MFGM) via multi-pathway and multi-target mechanisms. As illustrated in Figure 2, these bioactive compounds not only directly modulate the gut microenvironment by regulating immune cell function, alleviating systemic inflammation, and mitigating oxidative stress to reinforce the intestinal barrier, but also induce the production of beneficial metabolites, specifically short-chain fatty acids (SCFAs) such as acetate, propionate, and butyrate. These metabolites and bioactive components further exert their effects through the bidirectional regulatory network of the “gut–bone” axis, influencing the dynamic equilibrium between osteoblasts and osteoclasts. Specifically, SCFAs (particularly butyrate) activate the Runx2/BMP-2 signaling pathway to promote osteoblast differentiation and bone formation while simultaneously suppressing excessive osteoclastogenesis via osteoblast inhibition and modulation of key signaling pathways such as RANKL/RANK, thereby effectively enhancing bone mineral density and preserving bone microstructural integrity. This chapter systematically reviews the chemical structures and physicochemical properties of major milk-derived bioactive components. Furthermore, it focuses on elucidating the molecular mechanisms by which these components intervene in bone metabolic homeostasis through the regulation of the gut microbiota-SCFA axis and the immune-inflammatory microenvironment, providing a solid theoretical foundation for the development of novel milk-based functional foods targeting bone health.

### 4.1. The Regulation of Milk-Derived Components on the Structure and Function of Intestinal Flora

Milk sources (including cow milk, camel milk, goat milk, horse milk, etc.) are rich in active ingredients such as proteins, milk fat globule membranes, minerals, and oligosaccharides, which are natural key factors that regulate the structure and function of intestinal flora [87,88]. Among them, LF can selectively inhibit the growth of pathogenic bacteria through a dual mechanism, while oligosaccharides act as selective prebiotics to promote the proliferation of beneficial bacteria such as Bifidobacterium, thereby jointly reshaping the intestinal microecological balance [89,90].

In terms of antibacterial mechanism, LF does not play a role through a single pathway, but indirectly inhibits the proliferation of iron-demanding pathogenic bacteria by relying on iron chelation; that is, it preferentially combines with free iron to form an iron-LF complex, depriving free iron sources necessary for the growth of pathogenic bacteria such as *Escherichia coli* and *Staphylococcus aureus*. At the same time, LF can be digested and cleaved into lactoferricin, which has stronger antibacterial activity in a gastric acid environment and can directly destroy the integrity of pathogen cell walls and membranes [91,92]. Gallo et al. [88] confirmed this mechanism in vitro. Iron-saturated bLF can not only effectively inhibit iron-demanding pathogens, but also enable iron-independent beneficial bacteria such as *Bifidobacterium* and *Lactobacillus* to gain competitive growth advantages. This study further found that iron-saturated LF treatment increased the α-diversity of gut microbiota in healthy individuals and enriched gut microbiota associated with healthy aging (such as *Coprococcus*, *Alistipes* and *Bifidobacterium*) [87].

In terms of prebiotic effects, oligosaccharides (including human milk oligosaccharides (HMOs) and bovine milk oligosaccharides (BMOs)) are used as selective fermentation substrates, and their mechanisms of regulating flora are more diverse. First of all, they are specifically utilized by *Bifidobacterium* (such as *Bifidobacterium longum*, *Bifidobacterium breve*, and *Bifidobacterium infantis*) to produce metabolites (mainly acetic acid), which form a microenvironment conducive to the growth of beneficial bacteria by reducing local pH, while indirectly inhibiting acid-intolerant pathogens [93]. Secondly, oligosaccharides can competitively bind to the adhesion sites of pathogenic bacteria in the stomach and small intestine through the molecular simulation/bait receptor mechanism, preventing pathogens from adhering to the surface of intestinal mucosal epithelial cells [94]. Finally, some studies have also found that oligosaccharides can up-regulate goblet cell-related gene expression and tight junction protein levels, thereby maintaining intestinal epithelial integrity and strengthening intestinal barrier function [95]. Lee et al. [96] showed that milk oligosaccharides could promote the maturation of intestinal epithelial cells, enhance the expression level of tight junction proteins, and further strengthen the intestinal barrier function. At the same time, they can up-regulate goblet cell-related genes in the gastrointestinal tract and maintain intestinal epithelial integrity and intestinal barrier function [97].

On the basis of the above direct regulation of bone metabolism, there is also a “gut–bone” axis bidirectional regulation pathway between intestinal microorganisms and bones. Metabolites of the microbiota, such as SCFAs and bile acids, can directly affect bone formation and bone resorption through receptor signaling pathways (such as FXR and TGR5) and immune regulation, and maintain bone metabolism balance [98,99]. In addition, gut microbiota can also affect bone mineral density by regulating amino acid metabolism such as valine and leucine [100]. Studies have shown that extracellular vesicles secreted by intestinal beneficial bacteria such as *Akkermansia muciniphila* (*AKK*) can act on bone tissue, alleviate the decrease of *AKK* levels caused by ovariectomy (OVX), and then increase bone mineral density and bone strength [101].

On the contrary, intestinal flora imbalance is an important cause of osteoporosis. Flora disorder can destroy the intestinal barrier, increase the levels of pro-inflammatory factors (TNF-α, IL-1β, IL-6), activate the RANKL/NF-κB pathway, and disrupt bone homeostasis [102]. The decrease in estrogen caused by OVX can lead to a decrease in the diversity of intestinal flora in rats, a significant decrease in the number of beneficial bacteria (such as *Bifidobacterium*), and a significant increase in the bone resorption rate [103]. Wang et al. [104] found that compared with the distilled water control group, the relative abundance of *bifidobacteria* in the intestinal tract of mice in the Bactrian camel milk intervention group increased from 5.90% to 8.00%, but the relative abundance of *lactobacilli* decreased from 4.60% to 3.00%. Xie et al. [105] also pointed out that osteoporosis is accompanied by a decrease in beneficial bacteria and an increase in harmful bacteria producing SCFAs, thus disrupting the balance between osteogenesis and osteoclastogenesis.

In summary, milk-derived active ingredients regulate bone metabolism and maintain bone health through the “gut–bone” axis by regulating the structure and metabolic function of intestinal flora.

### 4.2. The Key Mediating Role of Short-Chain Fatty Acids (SCFAs)

Short-chain fatty acids (SCFAs) are the most important key mediators in the regulation of bone metabolism by intestinal flora. SCFAs are the main metabolites produced by intestinal flora by fermenting dietary carbohydrates (such as dietary fiber, oligosaccharides, etc.) [106], mainly including acetic acid, propionic acid and butyric acid, and the concentration ratio of the three is about 3:1:1 or 6:2:2 [107]. SCFAs play multiple roles in the regulation of bone metabolism. On the one hand, they maintain the integrity of the intestinal barrier and regulate the intestinal immune inflammatory response; on the other hand, they act directly on bone tissue, inhibit osteoclast differentiation, reduce bone resorption, promote osteoblast proliferation and differentiation, and enhance bone formation, thereby maintaining “Gut–bone” axis homeostasis and protecting bone health.

In terms of inhibiting osteoclast activity, SCFAs mainly play a role in two ways. One is to activate the GPR41 and GPR43 receptors on the surface of osteoclasts, reduce the production of inflammatory factors, and thus block osteoclast differentiation; secondly, butyric acid down-regulates the expression of bone resorption-related genes by inhibiting histone deacetylase (HDAC) activity [108]. Han et al. [46] confirmed that SCFAs protect cartilage and delay the progression of osteoarthritis by activating GPR41/43/109 A and HDAC pathways and reducing the expression of TNF-α, IL-1β and MMPs. In addition, propionic acid and butyric acid can also down-regulate the expression of key osteoclast-related genes such as TRAF6 and NFATc1 by affecting the oxidative phosphorylation process of osteoclasts [109]. In terms of promoting bone formation, acetate can stimulate the Wnt/β-catenin signaling pathway by activating the GPR43/GPR41 receptor to enhance osteogenic differentiation and bone formation; butyrate promotes bone formation by up-regulating the expression of osteogenesis-related genes (such as Runx2) and down-regulating osteoclast regulatory factors (such as NFATc1) [110].

### 4.3. Repair the Intestinal Barrier and Reduce Systemic Inflammation

The intestinal barrier is a key physiological barrier for the body to defend against the invasion of harmful substances from the outside world. Its structural integrity and functional stability are essential for the maintenance of systemic homeostasis [111]. When the intestinal barrier is damaged due to factors such as eating disorders, flora imbalance, oxidative stress or immune abnormalities, it will cause “intestinal leakage” [112]. The ways to repair the intestinal barrier mainly include up-regulating the expression levels of tight junction proteins (ZO-1, Occludin, Claudin) and regulating SIRT1/NRF2, TLR2/NF-κB and other related signaling pathways, thereby restoring the balance of intestinal flora.

Milk-derived active ingredients show potential value in intestinal barrier repair. Studies have shown that LF can effectively protect the intestinal immune barrier by regulating the TLR2/NF-κB/MLCK signaling pathway and inhibit excessive intestinal inflammation by reducing the levels of inflammatory factors such as IL-1β, IL-6 and TNF-α [113]. In addition, as previously mentioned, SCFAs also play an important role in maintaining the integrity of the intestinal barrier. Yue et al. [114] showed that *L. rhamnoides* significantly increased the content of three SCFAs, regulated the level of autophagy, repaired the mechanical and immune barrier of intestinal mucosa, and effectively improved 5-FU-induced intestinal inflammatory injury and intestinal barrier dysfunction. The above studies provide a theoretical basis for the bone protection of milk-derived active ingredients by repairing the intestinal barrier. Therefore, targeting the intestinal barrier may be another important way for milk-derived functional components to regulate bone health.

This section systematically elaborates on the systemic pathways through which milk-derived bioactive components indirectly regulate bone metabolism via the “gut–bone” axis. Bioactive components such as milk proteins, milk fat globule membrane, minerals, and oligosaccharides reshape the gut microbiota through dual mechanisms: LF selectively inhibits pathogenic bacteria and promotes the proliferation of beneficial bacteria such as *Bifidobacterium* by depriving pathogens of essential free iron through iron sequestration and by disrupting pathogen membranes via lactoferricin generated upon cleavage; oligosaccharides, acting as selective prebiotics, maintain intestinal barrier integrity through mechanisms including fermentation-derived acid production that lowers pH, molecular mimicry that blocks pathogen adhesion, and upregulation of goblet-cell and tight-junction protein expression. On the basis of this microbiota, the bidirectional regulatory pathway of the “Gut–bone” axis is established to operate: microbial metabolites, particularly SCFAs and bile acids, act directly on bone tissue through receptor signaling pathways such as GPR41/43, HDAC, FXR, and TGR5 as well as through immunomodulation, inhibiting osteoclast differentiation (downregulating TRAF6 and NFATc1), promoting osteogenic differentiation (activating Wnt/β-catenin and upregulating Runx2), while simultaneously repairing the intestinal barrier and attenuating systemic inflammation (downregulating IL-1β, IL-6, and TNF-α), thereby maintaining the osteoblast–osteoclast balance. Conversely, gut dysbiosis disrupts the intestinal barrier and activates the RANKL/NF-κB pathway, representing an important predisposing factor for osteoporosis. In summary, by regulating the composition and metabolic functions of the gut microbiota, milk-derived bioactive components bidirectionally regulate bone metabolism through the “gut–bone” axis, constituting another important pathway for bone health regulation distinct from direct cellular signaling.

## 5. Extraction and Identification Technology of Milk-Derived Functional Components

The physical properties of different milk-derived active ingredients are different, and the preparation and extraction methods for each are also different. For active peptides such as LF, OPN, MFGM and CPP, different exclusive extraction techniques have been developed. Active proteins are mainly extracted by ion exchange chromatography, centrifugation, heparin affinity chromatography, the precipitation method, and membrane separation technology. At the same time, they are combined with high-performance liquid chromatography, isoelectric focusing and other auxiliary technologies, which can significantly improve the recovery rate and product purity of the target active components. In the preparation of active peptides, the hydrolysis efficiency of the enzyme can also be significantly improved by optimizing the enzymatic hydrolysis conditions (pH, temperature, enzyme substrate concentration, etc.) to improve the recovery rate of the target active peptide. Additionally, by combining precipitation, chromatography and supercritical-assisted technology, a more refined extraction of casein phosphopeptides and casein glycomacropeptides can be completed. The specific extraction processes and characteristics of different dairy-derived bioactive components are summarized in Table 1. Therefore, selecting the appropriate extraction and preparation process according to the physical and chemical properties of different milk-derived active ingredients is key to achieving efficient separation and purification.

### 5.1. Separation and Purification Technology of Milk-Derived Active Protein

#### 5.1.1. Separation and Purification of Lactoferrin

LF is an iron-binding glycoprotein with a molecular weight of about 77 kDa and an isoelectric point of about 8.5. Its alkaline isoelectric point and unique iron-binding properties provide a chemical basis for the design of various separation techniques [128]. The extraction and purification process of LF mainly involves two stages: rough separation and fine purification. Traditional extraction techniques include fractionation and precipitation (ammonium sulfate or ethanol precipitation), ion exchange chromatography, size exclusion chromatography, affinity chromatography, ultrafiltration, dialysis, and high-performance liquid chromatography [64].

LF is positively charged at neutral pH and can bind to SP-Sepharose, CM-Sepharose, and other cation exchange media [129]. Most of the acidic proteins in whey (such as β-lactoglobulin, pI ≈ 5.7; α-lactalbumin, pI ≈ 4.8 [130]) are negatively charged or have a net charge close to zero under the same conditions and are removed by flow-through or low-salt washing to achieve the separation and purification of LF and whey proteins. In addition, under the pH 6.6~7.0 conditions, the strong cation exchange resin synthesized by inverse suspension copolymerization can selectively adsorb LF in four different whey types by electrostatic interaction, and the adsorption efficiency is as high as 90% [131].

Membrane separation technology is mainly used as the pretreatment and concentration step of LF extraction. The combination of membrane separation technology and cation exchange chromatography can efficiently extract LF while ensuring the structural integrity of the protein. For example, after the raw materials are treated by ultrafiltration and dialysis, and then adsorbed by cation exchange resin, LF products can be obtained from skim milk [115]. Wang et al. [116] developed an aptamer affinity column (AAC) coupled with the high-performance liquid chromatography (HPLC) method for the extraction and detection of LF, which can achieve accurate and efficient separation and purification. In addition, heparin affinity chromatography combined with reversed-phase high-performance liquid chromatography–ultraviolet detection (HPLC/UV) can also efficiently extract and detect unmodified bLF [117]. Mahala et al. [118] used a novel pH-dependent isolation and identification method to extract LF. This method utilizes the isoelectric point characteristics of camel milk by regulating pH throughout the process, thereby retaining the active ingredients of LF. At present, cation exchange chromatography is commonly used in industry. Its yield can reach 90–98%, and the extraction effect is good.

#### 5.1.2. Isolation and Purification of Osteopontin

In view of the important role of OPN in bone health regulation, it is very important to establish an efficient separation and purification method for its research and application. At present, the separation and purification of OPN mainly depend on its unique physical and chemical properties. As a highly phosphorylated acidic calcium-binding glycoprotein, OPN has a strong negative charge under acidic conditions and a high affinity for calcium ions. These characteristics provide a theoretical basis for its separation and purification [132]. Specifically, OPN can be strongly bound to anion exchange media such as DEAE-Sepharose CL-6B under acidic pH conditions, thereby achieving better enrichment [119]. Ravi et al. [133] confirmed that the recovery rate of OPN could be maintained at 80% by anion exchange chromatography under optimal adsorption and resin washing conditions, thus achieving efficient preparation of OPN. Wang et al. [120] also pointed out that the isoelectric point of OPN was determined by polarity reversal capillary isoelectric focusing to complete the characterization of its physical and chemical properties. They then combined this with DEAE-Sephacel or a strong anion exchange HPLC column for retention characteristics analysis. This approach can achieve preliminary identification of OPN, thus significantly improving the extraction rate of OPN. In addition, Chen et al. [134] found that the enrichment of low-abundance OPN in milk by preparative reciprocating free-flow isoelectric focusing (RFFIEF) technology can greatly improve the enrichment efficiency of the target protein by pretreating large quantities of milk samples. Therefore, anion exchange chromatography is commonly used in the industrial preparation of OPN due to its simple operation and good separation effect, and has good market prospects.

#### 5.1.3. Separation and Purification of Milk Fat Globule Membrane

MFGM is a three-layer phospholipid-protein membrane structure that encapsulates milk fat globules. The appropriate extraction process is crucial for subsequent research and application. Significant progress has been made in the separation and purification of MFGM, and its key process includes four steps: fat globule separation, cream washing, MFGM release, and collection and purification [33]. Zhao et al. [121] used butter whey as raw material to enrich polar lipids through microfiltration and ultrafiltration coupling technology, and the phospholipid content of the extract was more than 42%. This method can be directly applied to the industrial preparation of MFGM.

In addition, MFGM with a complete structure can be efficiently separated and enriched from two industrial by-products of cheese and butter whey by differential centrifugation and ultracentrifugation [122]. Pan et al. [123] showed that MFGM could be better enriched by 1.4 μm ceramic microfiltration combined with centrifugation after adjusting pH to 4.8. At the same time, lipids in MFGM can also be quantified and characterized by high-performance liquid chromatography–mass spectrometry (HPLC-MS) detection [135]. Verma et al. [136] found that after acrylic acid functional modification of the PVDF membrane, the modified PVDF membrane has the dual effects of size screening and charge adsorption, which can specifically adsorb and intercept negatively charged MFGM and its related components. Therefore, the membrane filtration method has become one of the most promising methods in the enrichment of MFGM due to its good retention of active ingredients and ease of large-scale production.

### 5.2. Preparation and Purification of Milk-Derived Bioactive Peptides

#### 5.2.1. Enzymatic Hydrolysis Preparation Technology

Protease is a kind of enzyme that can catalyze the hydrolysis of protein. It is one of the key factors that determine the activity of peptides. It mainly functions by degrading proteins into small molecular peptides or amino acids. According to the cleavage site and specificity, proteases can be divided into endopeptidases (such as trypsin, chymotrypsin) and exopeptidases (such as aminopeptidase, carboxypeptidase) [137]. The endopeptidase mainly acts on the inside of the peptide chain to generate a polypeptide fragment with a larger molecular weight; the exopeptidase gradually releases small peptides or free amino acids from the end of the peptide chain. In addition, according to the optimal pH, proteases can be divided into acid proteases, alkaline proteases and neutral proteases [138].

The optimization of enzymatic hydrolysis process mainly focuses on key parameters such as temperature, pH, time, substrate concentration, and enzyme/substrate ratio (E/S), which directly affect the yield and functional properties of active peptides. Temperature can regulate enzyme activity by changing the spatial conformation and substrate structure of the enzyme. In the range of 30–45 °C, the antioxidant and antibacterial activities of the ultrafiltration peptide components gradually increased with increasing temperature. When the temperature further increased to 45–55 °C, the peptide activity decreased significantly [139]. Yang et al. [140] found that the milk protein peptide hydrolysate after microwave pretreatment at 300 W showed good antioxidant stability, and its peptide content increased with the increase of microwave power, reaching a maximum at 400 W. In addition, the optimization experiment of SGM enzymatic hydrolysate showed that the ACE inhibitory activity increased first and then decreased with changes in temperature, E/S and pH, indicating that there is an optimal range of action among the three factors [141]. In summary, by optimizing the enzymatic hydrolysis conditions, the hydrolysis efficiency can be significantly improved in a relatively mild reaction environment, thereby establishing a stable and efficient milk-derived active peptide preparation process.

#### 5.2.2. Preparation of Active Peptide

As a natural mineral absorption factor, the basic preparation of active peptides is still based on the enzymatic hydrolysis of alkaline protease and trypsin, combined with a variety of efficient separation techniques to achieve the separation and identification of casein phosphopeptides (CPP) with high purity and high calcium-binding activity. At present, the commonly used separation methods mainly include gel filtration chromatography, reversed-phase high-performance liquid chromatography and capillary electrophoresis [142].

The traditional CPP enrichment process mostly uses calcium salt, barium salt, rare earth ion selective precipitation, as well as hydroxyapatite, ion exchange and ERLIC chromatography for rough separation. In recent years, metal affinity materials such as IMAC, MOAC, TiO_2_, functional magnetic nanofillers and two-dimensional liquid chromatography have also been widely used in the fine purification of CPP [142]. Zhu et al. [126] showed that the molecular structure of CPP treated by supercritical CO_2_-assisted atomization (SAA) technology remained intact, and the antioxidant activity was not significantly affected. Under different calcium ion concentration conditions, high-purity active products can be obtained by the calcium–ethanol precipitation method. Based on the calcium-ethanol fractional precipitation process, Low et al. [127] achieved the fractional separation of CPP-calcium chelates with different calcium chelating abilities in 55% ethanol system by gradually increasing the amount of Ca^2+^.

Casein glycomacropeptide (CGMP) is mainly derived from the enzymatic hydrolysate of κ-casein. After enzymatic release, it can be separated and purified by precipitation, ultrafiltration and ion exchange chromatography according to its unique physical and chemical properties. Panchal et al. [124] found that CGMP is soluble in a specific concentration of trichloroacetic acid (TCA) solution, and most of the miscellaneous proteins can be precipitated by adding TCA, while CGMP is retained in the supernatant, thus achieving initial enrichment. In addition to selective precipitation, ion exchange and gel filtration chromatography can be combined to further improve purity. Nakano et al. [125] used ultrafiltration and deproteinization pretreatment to remove non-GMP components in whey and then prepared CGMP from bovine whey by batch anion exchange technology with chitosan as the adsorbent at pH 3.0. This method is simple and low cost. At present, ultrafiltration is widely used in industrial production because of its simple process, and ion exchange chromatography plays a key role in the preparation of high-purity CGMP samples.

## 6. Development and Application Challenges of Milk-Derived Bone Health Functional Products

As mentioned above, milk-derived functional components (such as CPP, LF, OPN, MFGM, etc.) can exert bone protective effects through direct action and indirect regulation of “gut–bone” axis. These mechanism studies have laid a theoretical foundation for the development of milk-based functional products for bone health. However, there are still many technical challenges, from mechanism research to product implementation. First, the active ingredients are easily degraded by the gastrointestinal tract after oral administration, and the bioavailability is low, which needs to be protected by steady-state and targeted delivery technologies. Secondly, existing research is mostly limited to the cell and animal levels and lacks high-quality human clinical trial evidence. Finally, different characteristic milk sources have variations in composition and functional characteristics, and their respective differentiation advantages need to be clarified. This section will focus on the above key challenges.

### 6.1. Steady-State and Targeted Delivery Technology of Active Ingredients

The core goal of steady-state technology is to provide physical or chemical protective barriers for active ingredients before they reach the target tissue, so as to overcome the degradation risks caused by gastrointestinal digestion, blood enzymatic hydrolysis, and the complex physiological environment in vivo. After oral administration, milk-derived active ingredients (such as CPP and LF) are easily degraded by digestive enzymes such as pepsin and trypsin and lose their function, resulting in extremely low bioavailability. Steady-state technology can significantly improve the stability of active ingredients by forming a protective layer on their surface through microencapsulation, nanoencapsulation, and other means.

Many studies have provided experimental evidence for the above strategies. Ponzini et al. [143] reported that nanoparticle-based delivery systems (such as liposomes, polymer nanoparticles and lipid nanoparticles) can encapsulate LF in nanocarriers, protect the stomach and achieve controllable release in the digestive tract, thereby further enhancing its oral bioavailability. Elmorshedy et al. [144] also confirmed that nanoparticle-microparticle delivery systems (NIMDs) have successfully achieved oral targeted delivery of colon cancer drugs mainly through the combination of lactoferrin nanoconjugates and polysaccharide-protein mixed polymers, which can effectively protect lactoferrin from gastrointestinal degradation and show application potential in the treatment of colon cancer.

### 6.2. The Lack of Clinical Evidence and the Design Difficulties of Human Intervention Trials

Although great progress has been made in the study of the mechanisms by which milk-derived functional components regulate bone health, the lack of human clinical evidence is still a key bottleneck restricting the development of functional products. First, because the dose of milk-derived functional components used in cell and animal experiments is much higher than the level that the human body can actually ingest through diet or supplements, and there is a lack of pre-clinical data between dose and effect, it is difficult to determine the dose suitable for the human body, which leads to the problem where a high dose causes body discomfort and a low dose cannot produce an effect [145].

Secondly, the lack of research on the synergistic regulation mechanisms between various milk-derived functional components limits the maximization of the efficacy of related products. The clinical trial cycle for bone health is generally long, making it difficult to observe statistically significant changes in short-term intervention trials. Such trials face practical challenges, such as high turnover rates and high testing costs. Gao et al. [146] showed that in order to control the burden of participation, short-term observations of behavioral changes (such as 1, 3 or 6 months after intervention) would not be considered. The experiments required 12–24 months to observe sustainable behavioral changes and clinical results. If a participant quits during the experiment, it becomes necessary to control the loss rate and keep it below 20%.

Finally, it is necessary to study high-risk populations for osteoporosis, but these people often take calcium, vitamin D or other anti-osteoporosis drugs at the same time, making it difficult to accurately distinguish the intervention effects of milk-derived active ingredients alone. Iuliano et al. [147] also showed that the doses of fortified calcium and vitamin D were much higher than the natural content of dairy products, so it was difficult to determine whether the natural active ingredients of dairy products contribute independently to bone health or whether the effects are solely due to exogenously fortified calcium and vitamin D. In some experiments, dairy products were only used as carriers to add other nutrients, further obscuring the independent intervention effects of dairy products themselves. If a healthy group is selected, the relevant interventions may not lead to significant improvements in indicators, resulting in insufficient overall effectiveness of clinical trials.

To overcome these challenges, several specific study designs can be considered for future research. First, a randomized crossover design could be adopted, in which participants serve as their own controls, thereby effectively controlling inter-individual variability and reducing the required sample size and trial duration. Second, given that BMD changes occur slowly and require long observation periods, bone turnover markers (such as procollagen type 1 *N*-terminal propeptide [P1NP] and *C*-terminal telopeptide of type 1 collagen [CTX]) could be used as earlier and more sensitive surrogate endpoints to evaluate intervention effects within a shorter time frame. Third, as noted above, to distinguish the independent effects of endogenous components in dairy products from those of exogenously fortified calcium and vitamin D, intervention groups should be designed to compare dairy products alone, fortified dairy products, and isocaloric non-dairy controls. Finally, to mitigate the confounding effects of concomitant anti-osteoporosis medication, future trials could enroll participants with relatively homogeneous baseline status or stratify and statistically adjust for medication use when analyzing bone health outcomes. Together, these strategies would improve the feasibility, sensitivity, and interpretability of clinical trials evaluating milk-derived functional components for bone health.

### 6.3. The Differentiation Advantages of Characteristic Milk Sources (Camel Milk, Sheep Milk, Horse Milk)

Camel milk is a unique characteristic milk source in arid and semi-arid areas. It has the characteristics of anti-inflammation, anti-oxidation and bacteriostasis, and is suitable for people with low immunity and milk allergies as a functional milk source [148]. The total protein content of camel milk is between 2.15% and 4.90% [149], and the protein content of camel milk in Xinjiang, China, is between 3.34% and 3.95% [150]. Camel milk fat globules are smaller and have a higher proportion of long-chain unsaturated fatty acids, which are more easily digested and absorbed by the human body [151]. Additionally, it does not contain β-lactoglobulin (the main milk protein allergen) [152], and the proportion of α-casein is low. The proportions of β-casein and κ-casein are close to those found in human milk [153], so allergenicity is low, and most people with lactose intolerance can consume it. Camel milk is rich in LF, lysozyme and immunoglobulin. The activity and content of lactoferrin in camel milk are much higher than those in cow milk and goat milk. Camel milk has strong stability and can effectively inhibit bacteria, resist viruses and enhance mucosal immunity [154]. The contents of calcium, iron and other minerals and vitamins in camel milk are also higher than those in cow milk, especially the content of vitamin C, which is 3–5 times that of cow milk [155]. In addition, a variety of bioactive components in camel milk have antibacterial, antioxidant and anti-inflammatory properties and are used in the medical field to assist in the treatment of diseases such as hepatitis, gastric ulcer and diabetes.

Compared with bovine milk, goat milk fat globules have a diameter of about 3.19–3.50 μm and have good digestion and absorption characteristics [156]. The proportion of β-casein in goat milk is the highest, reaching about 54.8%, while the content of α-casein is much lower than that in bovine milk, and allergenicity is greatly reduced [157]. The calcium content (193 mg/100 g) and phosphorus content (158 mg/100 g) of goat milk are higher than those of bovine milk (calcium 117 mg/100 g, phosphorus 93 mg/100 g), which can directly promote the process of bone mineralization [158]. The calcium–phosphorus ratio of goat milk is higher than that of cow milk, and the bioavailability of calcium is higher, which has a positive effect on bone health [159]. Therefore, goat milk is particularly suitable for the development of infant formula, functional milk powder for middle-aged and elderly individuals, and characteristic dairy products for gastrointestinally sensitive people. Studies have found that goat-milk-based infant formula can significantly increase the mRNA expression levels of intestinal tight junction proteins (occludin and claudin), and the effect is better than that of cow milk [160].

The nutritional composition and functional characteristics of horse milk are very similar to those of human milk, making it less likely to cause allergic reactions. It can be used as an effective substitute for milk. It is significantly superior to cow and goat milk in terms of essential amino acid composition and protein biological value. Compared with bovine and goat milk, horse milk protein content is 1.4–3.2 g/100 mL, lactose 5.6–7.2 g/100 mL, and fat 0.3–4.2 g/100 mL, making it closer to human milk [149]. The fat content of horse milk is significantly lower than that of human milk and cow milk, and the proportion of unsaturated fatty acids is closer to that of human milk. The whey protein content is also close to that of human milk and higher than that of cow milk [161]. Horse milk is easy to digest and absorb. Its functional characteristics include bacteriostasis, antiviral efficacy, immunity enhancement, regulation of intestinal flora, and anti-inflammation and anti-oxidation effects. It also has the advantages of being organic, safe and nutritionally balanced. It is a high-quality characteristic functional milk resource.

## 7. Conclusions

This article focuses on the dual mechanisms—both direct and indirect—by which functional components derived from milk (CPP, LF, OPN, MFGM) and minerals regulate the osteoblast-osteoclast balance, as well as the current status of extraction and purification technologies and product development applications. Milk-derived functional components can target and modulate the biological activities of osteoblasts and osteoclasts. On one hand, LF mediates the dynamic balance between bone formation and bone resorption through the activation of key signaling pathways such as Wnt/β-catenin and MAPK; CPP, in combination with calcium and phosphorus, synergistically promotes osteogenic activity. On the other hand, LF and MFGM can regulate the structure and function of the gut microbiota via the “gut–bone” axis, promoting the production of SCFAs and lowering intestinal pH, thereby significantly enhancing calcium bioavailability. Simultaneously, they indirectly mediate bone metabolic regulation by modulating the bone immune microenvironment.

In terms of milk source extraction technology, membrane separation, ion exchange chromatography, and other technologies have been widely used in the separation and preparation of various milk source active proteins. The operation is simple, and the process system is relatively mature. However, different milk proteins face technical challenges such as protein degradation and inactivation in the acidic environment of the stomach, and low bioavailability. Therefore, steady-state and targeted delivery technology still needs further study and analysis of the synergistic mechanism between milk source components, as well as the optimization and improvement of key technical problems. At present, the research on the regulation of bone health by milk-derived functional components still has the following deficiencies: First, the synergistic effect of different milk source components on the bone health regulation mechanism is not clear, in addition to many other issues; second, there is a lack of clinical evidence (mostly limited to cell and animal experiments) and a serious lack of high-quality human clinical trial data support; third, research on characteristic milk sources lags behind (such as cow milk, camel milk, horse milk and goat milk), and other functional components are insufficiently explored.

Although progress has been made in the research on milk-derived functional components in the field of bone health, the strategic focus of future research needs to shift further from “mechanism validation” to “clinical application.” However, three key bottlenecks remain to be addressed in this regard. First, the regulatory effects on signaling pathways observed at the cellular and animal levels should be translated into differentiated precision nutrition interventions targeting key populations, including postmenopausal women, the elderly, and patients with osteoporosis. Second, priority should be given to developing stabilization technologies such as nano-encapsulation and intestine-targeted release, so that product forms can evolve from conventional dietary supplements toward medically oriented nutritional products with more clearly defined functional indications. Third, the limitations of single-component research must be overcome by leveraging the synergistic effects among different milk-derived functional components, thereby providing a theoretical foundation for the scientific formulation of composite preparations.

To translate these mechanistic insights into clinical benefit, we propose a stepwise roadmap for future research. First, well-designed human RCTs with sufficient duration and adequate sample sizes are urgently needed to validate the bone-protective effects of milk-derived components across key populations such as postmenopausal women, the elderly, and osteoporosis patients. Second, integrating multi-omics approaches (e.g., genomics, transcriptomics, metabolomics, and metagenomics) will help deconvolute the complex regulatory networks through which milk-derived components influence the osteoblast-osteoclast balance. Third, given the central role of the gut–bone axis, future studies should move toward personalized nutrition based on gut microbiota profiles, identifying microbiota-derived biomarkers to stratify responders and enable tailored precision interventions. Together, these efforts will ultimately strengthen the evidence base for developing safe and effective milk-derived functional foods for bone health.

In summary, milk-derived bioactive components, as natural and safe dietary ingredients for improving bone health, possess significant scientific research value and application potential. Nevertheless, further in-depth studies are still needed to elucidate the synergistic mechanisms among different milk-derived bioactive components and to improve novel technologies for the stable delivery of bioactive proteins, thereby solidifying the scientific theoretical foundation for the development of bone-protective functional foods derived from milk.

## Figures and Tables

**Table 1:** Comparison of separation and purification processes and technical characteristics of major dairy-derived bioactive substances.

	Target Component	Extraction Methods	Merits	Shortcomings	References
Separation and Purification Technology of Bioactive Proteins from Whey	Lactoferrin	1. Cation exchange resin 2. Heparin affinity chromatography and reversed-phase high-performance liquid chromatography (RP-HPLC) detection. 3. pH-dependent separation and identification.	1. High specific adsorption capacity. 2. High binding capacity, high recovery rate and excellent stability. 3. Suitable for large-scale production and industrial application.	1. Multiple purification steps are required to obtain high-purity products. 2. The coupling process of heparin ligand is complicated, which requires expensive heparin ligands and special chromatographic media. 3. Precise pH control is demanded, leading to high operational difficulty.	[115,116,117,118]
	Osteopontin	1. Anion exchange. 2. Preparative recycling free-flow isoelectric focusing (RFFIEF) technology.	1. Highly compatible with the acidic properties of OPN and achieves favorable separation performance. 2. Large processing throughput, fast separation speed and mild liquid phase environment.	1. High requirements for sample pretreatment and limited resolution. 2. Risk of isoelectric precipitation and loss of hydrophobic proteins.	[119,120]
	Milk fat globule membrane	1. Coupled microfiltration and ultrafiltration. 2. Differential centrifugation combined with ultracentrifugation. 3. High-performance liquid chromatography-mass spectrometry detection.	1. Scalable, mild operating conditions and high concentration efficiency. 2. Simple operation and a well-established gold-standard method. 3. High detection sensitivity, suitable for the analysis of complex samples and high accuracy.	1. Membrane fouling issues, strict precise parameter control requirements and relatively high cost. 2. Limited separation purity, high energy consumption and expensive equipment. 3. High cost and complicated sample pretreatment.	[121,122,123]
Preparation and purification of milk-derived bioactive peptides	Casein glycomacropeptide	1. Precipitation method. 2. Ion exchange chromatography.	1. High-purity products can be obtained with simple processes and controllable glycosylation degree. 2. High recovery rate, high purity and good retention of glycosylated GMP.	1. Extremely low recovery rate, severe loss of non-glycosylated GMP and massive raw material waste. 2. Complicated operation requiring multi-stage separation, high resin cost and troubles in eluent treatment.	[124,125]
	Casein phosphopeptides	1. Supercritical CO_2_-assisted atomization (SAA). 2. Calcium-ethanol fractional precipitation process. 3. Capillary electrophoresis technology.	1. Significantly improved dissolution rate and mild process conditions. 2. Improved bioavailability, greatly enhanced product purity and mature industrial applicability. 3. Powerful separation capacity, high analytical sensitivity and wide application range.	1. Strict requirements for process parameters, high equipment cost and complicated operation. 2. Yield loss, long production cycle and complex multi-stage separation procedure. 3. Relatively high equipment cost, limited detectable wavelengths, and only applicable to the detection of high-purity CPP preparations.	[126,127]

## Data Availability

No new data were created or analyzed in this study. Data sharing is not applicable to this article.
